# Supplementary material for: Assessing the knowledge, attitudes, practices, and perspectives of stakeholders of the deworming program in rural Rwanda
Source: PLoS Negl Trop Dis. 2023 Aug 8;17(8):e0010759. doi: 10.1371/journal.pntd.0010759 (PMC10437854; doi:10.1371/journal.pntd.0010759)
Supplement: S1 Appendix — (DOCX) [file pntd.0010759.s001.docx]

# S1_Appendix: Quantitative data collection tool

**Demographics:**

| **Age:** |  |
| --- | --- |
| **Sex:** | □   Male            □ Female |
| **Address:** | District:              Sector:             Cell:                     Village: |
| **Profession/Occupation:** | □ CHW □ Teacher |
| **Highest level of education attained:** | □ None       □ Primary       □ Secondary     □ University  □ Vocational training only       □ Literacy classes only |
| **Marital status:** | □ Single      □ Married       □ Divorced       □ Widowed |
| **Years of experience as a teacher/CHW:** |  |
| **Were you trained on deworming?** | □ Yes      □ No |
| **Year when you were last trained on deworming:** | □ 2016      □ 2017      □ 2018      □ 2019   □ 2020      □ 2021 |

**KAP for CHWs and teachers**

**Knowledge**

| **Section 1. Schistosomiasis** | **Yes** | **No** | **I don’t know** |
| --- | --- | --- | --- |
| - Schistosomiasis is treatable at the health center | □ | □ | □ |
| - You can transmit schistosomiasis from open defecation | □ | □ | □ |
| - Swimming in contaminated water can lead to schistosomiasis | □ | □ | □ |
| - Washing clothes in contaminated water can lead to schistosomiasis | □ | □ | □ |
| - You can get schistosomiasis from eating unripe fruits | □ | □ | □ |
| - You can contract schistosomiasis through sexual intercourse | □ | □ | □ |
| - Diarrhea is a symptom of schistosomiasis | □ | □ | □ |
| - The most common symptom of schistosomiasis is fever | □ | □ | □ |
| - Pain during urination is a symptom of schistosomiasis | □ | □ | □ |
| **Section 2: Soils Transmitted Helminths (STH)** | **Yes** | **No** | **I don’t know** |
| - You can acquire STH from walking bare foot | □ | □ | □ |
| - If you were cured from STH, you can never get it again. | □ | □ | □ |
| - Diarrhea is a symptom of STH | □ | □ | □ |
| - Fever is the most common symptom of STH | □ | □ | □ |
| - STH is hereditary | □ | □ | □ |
| - You can acquire STH through direct skin contact | □ | □ | □ |
| - You can get STH from drinking and eating contaminated water and food | □ | □ | □ |

**Attitudes**

| **Section 1: Schistosomiasis** | **Strongly agree** | **Agree** | **Disagree** | **Strongly disagree** |
| --- | --- | --- | --- | --- |
| - Deworming is helpful in treating schistosomiasis | □ | □ | □ | □ |
| - The people in my community are at high risk of acquiring schistosomiasis | □ | □ | □ | □ |
| - I am an important contributor to the prevention of schistosomiasis in my community | □ | □ | □ | □ |
| - The frequency of mass drug administration against schistosomiasis in schools/community is enough | □ | □ | □ | □ |
| - Schistosomiasis can be best treated by traditional healers | □ | □ | □ | □ |
| **Section 2: STH** | **Strongly agree** | **Agree** | **Disagree** | **Strongly disagree** |
| - Deworming is helpful in treating STH | □ | □ | □ | □ |
| - The people in my community are at high risk of acquiring STH | □ | □ | □ | □ |
| - I am an important contributor to the prevention of STH in my community | □ | □ | □ | □ |
| - The frequency of mass drug administration against STH in schools/community is enough | □ | □ | □ | □ |
| - STH can be best treated by traditional healers | □ | □ | □ | □ |

**Practices**

|  | **Never** | **Rarely** | **Often** | **Always** |
| --- | --- | --- | --- | --- |
| - I encourage children/ people to wash their hands | □ | □ | □ | □ |
| - I wash my hands before eating | □ | □ | □ | □ |
| - I drink untreated water | □ | □ | □ | □ |
| - I swim in rivers/ lakes | □ | □ | □ | □ |
| - I wash clothes or utensils in open water source | □ | □ | □ | □ |
| - What is the common source of drinking water you use? | □ River/Lakes  □ Tap water  □ Boreholes  □ Others, please specify___________ | | | |
