# Supplementary material for: Assessing the knowledge, attitudes, practices, and perspectives of stakeholders of the deworming program in rural Rwanda
Source: PLoS Negl Trop Dis. 2023 Aug 8;17(8):e0010759. doi: 10.1371/journal.pntd.0010759 (PMC10437854; doi:10.1371/journal.pntd.0010759)
Supplement: S2 Appendix — (DOCX) [file pntd.0010759.s002.docx]

**S2_Appendix: Qualitative data collection tool**

**Local leaders’ qualitative interview questions**

1. As a village leader, can you describe your experience working in the decentralized deworming program?
2. As village leader, what challenges, if any, have you faced while working in the decentralized deworming program?
3. What reactions have you observed or received from your community on the decentralized deworming program?
4. What interventions, apart from deworming, have you implemented or tried to implement in your community to address/combat intestinal worms?
5. What recommendations do you have on how the decentralized deworming program can be improved?
6. Is there anything else you would like to share with us regarding this program that might not have been discussed during this interview?
