# Supplementary material for: Assessing the knowledge, attitudes, practices, and perspectives of stakeholders of the deworming program in rural Rwanda
Source: PLoS Negl Trop Dis. 2023 Aug 8;17(8):e0010759. doi: 10.1371/journal.pntd.0010759 (PMC10437854; doi:10.1371/journal.pntd.0010759)
Supplement: Attachment — Submitted filename: Comments on PLOS Review.docx [file pntd.0010759.s004.docx]

Comments on PLOS Review:

**Introduction**

- Line 71: is citation 13 for both statements?
- Line 75: missing a word before citation 15, “children infected with” what?
- Line 76: remove comma, also a very wide range?
- Line 79: “in conjunction” rather than “conjunct”
- Line 91: “and CHWs,”

**Methods**

- Line 109/110: possible duplication of “Sample”?
- Line 118: would it be possible to give some examples of what roles “local leaders” perform in the community? (i.e. civil, religious, etc.)
- Line 144: I believe this is the first mention of “cells”. There is a definition given later, all the way down in line 316 of the results. Please move this description to line 144 where “cell” is first used.
- Line 149: Please give a short description of what is meant by “theoretical saturation”, and a reference for “purposive sampling”.
- Line 158: “Informed **consent** **was** obtained”
- Line 166: “Informed **consent**”
- Line 174: a citation for the Likert scale may be appropriate <https://psycnet.apa.org/record/1933-01885-001>
- Line 186: What was the pre-testing mentioned here? Is this internal with the research team or were potential respondents shown the draft survey?
- Line 201: A reference for Fisher’s exact test may be appropriate <https://link.springer.com/referenceworkentry/10.1007/978-3-642-04898-2_253>
- Line 202/3: From this, it is hard to understand what attitude and practice categories really are. Describing the possible categories in the methods may be useful.
- Line 206: The statement says that all statistical tests were performed with a p-value of 0.05. This is confusing, is it perhaps the case that the authors mean that all results of the statistical tests with a p-value <0.05 were deemed to be significant?
- Good description of the translation/coding in lines 207-210, maybe a citation for Dedoose software would be appropriate.
- Line 212: Does the University of Global Health Equity IRB have an identification number or code?

**Results**

- Table 1: It may be more clear to indicate that “Education level” is the “Highest Level of Education Attained”. Otherwise some may be confused that only 1 Teacher had a primary level of education while 314 had a secondary level of education.
- Table 1: Is “Training in deworming” the same as “training on worm infections”? It is a little confusing to see them presented one above the other as though they are maybe the same.
- Table 1: I am a little confused about the p-values in table 1 that are seemingly comparing across three columns and 2 rows at the same time with the same value. For example, what is the p-value 0.058 in the “Year when they received training on worm infections” actually comparing? It doesn’t seem that it should be exactly the same for both of the year categories.
- Table 3: The format makes it seem as though maybe only strongly agree and strongly disagree results are presented, but given that the total is 100% I assume the parentheses indicate these are combined across strongly/regular agreement. If they are to be combined, I would recommend changing the column headings to make this more clear, i.e. Agreement (strong/regular) and Disagreement (strong/regular), but I think it would be best to present both strong/regular and then combined as separate columns.
- Table 5: The second poor water source option is a little confusing to a reader not from this region. Is Swamps/Rock indicative of surface water from swampy/rocky areas? Or is “Rock” meant to indicate groundwater from a spring thatcomes out of rocks?

**Discussion**

- Line 394/395 is “knowledge about symptoms and treatment” from the Ivory coast study? If so, it would be clearer to combine this with the previous sentence/citation.
- It would be interesting and useful to see further research or an addition to this study that indicates some method of improving/strengthening the program curriculum (as recommended in line 399). Perhaps conjecture in this direction could be made in the limitations/further study section at the end of the discussion?
- Line 438 says that a significant portion (35%) reported drinking untreated water, with no p-value reported. Please include the significant p-value (i.e. (35%, p<0.xx)).
