# Supplementary material for: Assessing the knowledge, attitudes, practices, and perspectives of stakeholders of the deworming program in rural Rwanda
Source: PLoS Negl Trop Dis. 2023 Aug 8;17(8):e0010759. doi: 10.1371/journal.pntd.0010759 (PMC10437854; doi:10.1371/journal.pntd.0010759)
Supplement: Attachment — Submitted filename: Response letter to reviewers. docx.docx [file pntd.0010759.s006.docx]

Dear Reviewers,

Thank you for your valuable comments and input which allowed us to improve our manuscript. Please find below a detailed list of all the amendments, with line references from the manuscript:

**Introduction**

- Line 71: is citation 13 for both statements? ***Yes, the citation was for both statements which were merged (Line 71)***
- Line 75: missing a word before citation 15, “children infected with” what? ***Yes, the missing word was added (line 76)***
- Line 76: remove comma, also a very wide range? ***Yes, the comma was removed***
- Line 79: “in conjunction” rather than “conjunct” ***This was corrected (line 80)***
- Line 91: “and CHWs,” ***This was corrected (line 92)***

**Methods**

- Line 109/110: possible duplication of “Sample”? ***Corrected, 110 was removed as it was a repetition (Line 110)***
- Line 118: would it be possible to give some examples of what roles “local leaders” perform in the community? (i.e. civil, religious, etc.) ***This was addressed (line 119)***
- Line 144: I believe this is the first mention of “cells”. There is a definition given later, all the way down in line 316 of the results. Please move this description to line 144 where “cell” is first used. ***This was addressed (line 146)***
- Line 149: Please give a short description of what is meant by “theoretical saturation”, and a reference for “purposive sampling”. ***This was addressed (line 151)***
- Line 158: “Informed **consent** **was** obtained” ***This was addressed (line 163)***
- Line 166: “Informed **consent**” ***This was addressed (line 171)***
- Line 174: a citation for the Likert scale may be appropriate. ***This was addressed (line 181)***

<https://psycnet.apa.org/record/1933-01885-001>

- Line 186: What was the pre-testing mentioned here? Is this internal with the research team or were potential respondents shown the draft survey? ***The pre-testing was external with a population with similar characteristics as the respondents (line 191)***
- Line 201: A reference for Fisher’s exact test may be appropriate ***This was addressed (Line 216)*** <https://link.springer.com/referenceworkentry/10.1007/978-3-642-04898-2_253>
- Line 202/3: From this, it is hard to understand what attitude and practice categories really are. Describing the possible categories in the methods may be useful. ***This was explained in the “Measures "section in the methods (line 196 for attitude and 202 for practices)***
- Line 206: The statement says that all statistical tests were performed with a p-value of 0.05. This is confusing, is it perhaps the case that the authors mean that all results of the statistical tests with a p-value <0.05 were deemed to be significant? ***Yes, this was clarified (line 221)***
- Good description of the translation/coding in lines 207-210, maybe a citation for Dedoose software would be appropriate. ***This was addressed (line 226)***
- Line 212: Does the University of Global Health Equity IRB have an identification number or code? ***We reached out to the university IRB, and they responded that they do not have an identification number, however they are ready to share documentation certifying of its operation. (Line 228)***

**Results**

- Table 1: It may be more clear to indicate that “Education level” is the “Highest Level of Education Attained”. Otherwise some may be confused that only 1 Teacher had a primary level of education while 314 had a secondary level of education. ***“Education level” was replaced by “Highest Level of Education Attained”***
- Table 1: Is “Training in deworming” the same as “training on worm infections”? It is a little confusing to see them presented one above the other as though they are maybe the same. ***“Training in deworming” was corrected to “training on worm infections”. Only those who reported that they had received training on worm infections were asked which year they had received the training.***
- Table 1: I am a little confused about the p-values in table 1 that are seemingly comparing across three columns and 2 rows at the same time with the same value. For example, what is the p-value 0.058 in the “Year when they received training on worm infections” actually comparing? It doesn’t seem that it should be exactly the same for both of the year categories. ***The P-value in the “Year when they received training on worm infections” (0.058) is comparing the number of CHWs and teachers who received training in the first-year category (2016-2018) to those who received training in the second-year category (2019-2021). The P-value is a comparison between two columns (CHWS and Teachers). The first column contains the total number of CHWs and teachers in each category.***
- Table 3: The format makes it seem as though maybe only strongly agree and strongly disagree results are presented, but given that the total is 100% I assume the parentheses indicate these are combined across strongly/regular agreement. If they are to be combined, I would recommend changing the column headings to make this more clear, i.e. Agreement (strong/regular) and Disagreement (strong/regular), but I think it would be best to present both strong/regular and then combined as separate columns. ***The column headings were changed as suggested to make it clearer: “(Strongly) agree” was replaced by “Agreement (strong/regular)” while “(Strongly) disagree” was replaced by “Disagreement (strong/regular)”***
- Table 5: The second poor water source option is a little confusing to a reader not from this region. Is Swamps/Rock indicative of surface water from swampy/rocky areas? Or is “Rock” meant to indicate groundwater from a spring that comes out of rocks?***“ Swamps/Rocks” was clarified as Swamps or Rocks, to signify participants who use water from either swamps or rocks. (The poor water sources were classified in two categories, the first being” Rivers or lakes while the second was “Swamps or Rocks”.***

**Discussion**

- Line 394/395 is “knowledge about symptoms and treatment” from the Ivory coast study? If so, it would be clearer to combine this with the previous sentence/citation. **T*he “knowledge about symptoms and treatment” finding mentioned on this line was not from the Ivory coast study, instead it was from our own study. This statement was clarified as follows: “Findings from our study showed that knowledge about symptoms and treatment of worm infections were low”. (Line 409)***
- It would be interesting and useful to see further research or an addition to this study that indicates some method of improving/strengthening the program curriculum (as recommended in line 399). Perhaps conjecture in this direction could be made in the limitations/further study section at the end of the discussion? ***We acknowledged the suggested limitation and added it to our suggestions for further research (line 468)***
- Line 438 says that a significant portion (35%) reported drinking untreated water, with no p-value reported. Please include the significant p-value (i.e. (35%, p<0.xx)). ***The statement was clarified: The word “significant” was previously used to mean a considerable number rather than a statistically significant one. (Line 454)***

**Comments within the document**

- Good title:

***Thank you***

**Abstract**

- This statement on Knowledge, attitude & practices is it referred to Rwanda alone or other countries in Sub-Sahara Africa? The investigators need to clarify this

***The statement was clarified, by adding “in Rwanda” (Line 23)***

- Other critical stakeholders such as the school age children and community members who are beneficiaries of the deworming program are missing in this study. Are they deliberately excluded from the study as this could potentially be a serious limitation of the study.

***The need to get beneficiaries perspectives was acknowledged in the limitations section of the study under discussion (Line 468)***

- The investigators should add that geographical and financial limitations are the major cause of resistance to deworming exhibited by caregivers

***" The geographical and financial limitations” mentioned were a limitation to accessing clean water rather than a cause of resistance to the deworming program by caregivers. (Line 30)***

**Author summary**

- Eradicate is too strong and should be replaced with eliminate, as schistosomiasis is being targeted for elimination as detailed in the WHO new Road Map target 2030

***The word “Eradicate” was replaced with “Eliminate” as suggested. (Line 42)***

**Results**

- The practices variables should be recasted to read: Encouraging children/people to wash their hands. The word I should be removed.

***This was done (Table 4)***

- Recast variables by removing the word I

***This was done. (Table 8)***

**Discussion**

- The reasons for indulgence in this practice could not be explained/ascertained.

***This is right, as mentioned in the paragraph, more research to understand the decision process leading to the behavior is needed. (Line 462)***

We are looking forward to your feedback!
