# Supplementary material for: Assessing the knowledge, attitudes, practices, and perspectives of stakeholders of the deworming program in rural Rwanda
Source: PLoS Negl Trop Dis. 2023 Aug 8;17(8):e0010759. doi: 10.1371/journal.pntd.0010759 (PMC10437854; doi:10.1371/journal.pntd.0010759)
Supplement: Attachment — Submitted filename: Review March 2023.docx [file pntd.0010759.s008.docx]

**Dear reviewer #1: we acknowledge your comments and thank you for your review throughout the process.**

**Dear reviewer #2: Thank you for your valuable comments and review. Please find below our comments in blue for your consideration:**

Methods

Reviewer #1: Yes, methods strong throughout the process.

Reviewer #2: The objectives of the study were clearly stated. The study design was found to be appropriate. The population was clearly stated but the study would have included school age children who are  one of the critical stakeholders and beneficiaries of deworming Programme. Their insights and perspectives on deworming activities are equally needed and will make important contributions in the study of this kind.  I think the sample size is adequate and equally addressed the hypothesis being tested.

Correct statistical analysis and variables were used to support the study.

There are no known ethical concerns as the study did not involve any invasive procedures.

**We addressed the question regarding including children in the review section titled “Summary and General Comments” below**

--------------------

Results


Reviewer #1: Yes, changes were made to have the tables be a little clearer to read and understand.

Reviewer #2: The analysis presented matched the analysis plan. The results are clearly presented.

The figures and tables are of sufficient quality.

--------------------

Conclusions
-

Reviewer #1: Yes, the conclusion is well written and clearly addresses limitations and use of these data for advancing the knowledge base.

Reviewer #2: The authors have to clearly describe and  highlight the conclusions emanating from this study.  They need to further describe the limitations of the analysis if any. I did not see in the manuscript, major recommendations and implications of the findings of the study to the National Deworming Programnme of Rwanda.  By  clearly describing how the outcomes of the study will impact on the Deworming Programme and may contribute to informed-decision making/policy change, this will bring to fore the public health relevance of the study.

**The recommendations and implications of the study are imbedded in the discussion section of the protocol as suggested by the PLOS neglected tropical disease publication guidelines website** <https://journals.plos.org/plosntds/s/submission-guidelines> **We formulated our manuscript based on these guidelines.**

**The limitations of the study were highlighted in the last paragraph of the discussion section in the manuscript.**
--------------------
Editorial and Data Presentation Modifications?

Reviewer #1: All concerns and comments from the first submission have been satisfactorily addressed.

Reviewer #2: I recommend that the minor revision  be done, and that the authors should carefully address the comments made in the draft manuscript.

**We reviewed the comments within the document and are sharing again the comments here below:**

**“**•	Good title:

**Thank you**

Abstract

•	This statement on Knowledge, attitude & practices is it referred to Rwanda alone or other countries in Sub-Sahara Africa? The investigators need to clarify this

**The statement was clarified, by adding “in Rwanda” (Line 23)**

•	Other critical stakeholders such as the school age children and community members who are beneficiaries of the deworming program are missing in this study. Are they deliberately excluded from the study as this could potentially be a serious limitation of the study.

**The need to get beneficiaries perspectives was acknowledged in the limitations section of the study under discussion (Line 468)**

•	The investigators should add that geographical and financial limitations are the major cause of resistance to deworming exhibited by caregivers

**" The geographical and financial limitations” mentioned were a limitation to accessing clean water rather than a cause of resistance to the deworming program by caregivers. (Line 30)**

Author summary

•	Eradicate is too strong and should be replaced with eliminate, as schistosomiasis is being targeted for elimination as detailed in the WHO new Road Map target 2030

**The word “Eradicate” was replaced with “Eliminate” as suggested. (Line 42)**

Results

•	The practices variables should be recasted to read: Encouraging children/people to wash their hands. The word I should be removed.

**This was done (Table 4)**

•	Recast variables by removing the word I

**This was done. (Table 8)**

Discussion

•	The reasons for indulgence in this practice could not be explained/ascertained.

**This is right, as mentioned in the paragraph, more research to understand the decision process leading to the behavior is needed. (Line 462)”**

--------------------

Summary and General Comments

Reviewer #1: (No Response)

Reviewer #2: My overall comment is that the authors should address the gaps highlighted in the manuscript. One of the key beneficiaries of deworming Programme which are school age children were left out of this study. The authors should find away to interview them and incorporate their views and perspectives into the finding.

**The authors agree that the perspectives of the community including children is important as they are the beneficiaries of the program. This was highlighted as a recommendation for further research which can be a separate study. This study focused on the implementers of the program as the findings will help to provide them with more capacity to educate the population. Results from this study will also help to understand the challenges they face while implementing the program.**

--------------------

**We are looking forward to your feedback!**

**Thank you.**
